# Aurora Observations from the Principality of Transylvania from the 16th to the 18th Century CE

**DOI:** 10.1007/s11207-021-01811-7

**Published:** 2021-05-03

**Authors:** Martin Stangl, Ulrich Foelsche

**Affiliations:** 1 Institute for Geophysics, Astrophysics, and Meteorology/Institute of Physics (IGAM/IP), NAWI Graz, University of Graz, Graz, Austria; 2 Wegener Center for Climate and Global Change (WEGC), University of Graz, Graz, Austria

**Keywords:** Solar activity, Auroral activity, Maunder minimum, Historical documents, Hungarian history

## Abstract

We focused on the period from about 1500 CE to 1800 CE and present a compilation of 78 different auroral sightings for the period from the geographical area of the former Principality of Transylvania, then part of the Kingdom of Hungary, and we give source quotations in English translation. Of the 78 potential aurorae, 23 are missing from the catalog of Rethly and Berkes (1963) and are introduced here for the first time into the scientific discourse on past solar activity. The region of Transylvania located around 46° northern latitude is a good geographical indicator for an auroral oval extending unusually far towards the Equator. The reports of seven celestial phenomena from Transylvania during the period of the Maunder minimum (1645 – 1715), which are considered genuine northern lights at a medium to very high probability level, suggest that even during this time of greatly reduced solar activity, aurorae penetrated down to near 45° latitude. Three of these potential aurorae, however, fall within the 18th century, when the Sun was already recovering from the deep minimum phase. Comparing the distribution of potential auroral sightings in Transylvania from the 16th to the 18th century clearly shows, in comparison with other aurora catalogs and with reconstructed solar activity, that high selectivity due to the historical-source situation (incomplete chronicles, lost reports, and lack of scientific interest on the part of chroniclers) makes statements about actual long-term distributions almost impossible. Furthermore, the catalog of Rethly and Berkes is shown to be rather incomplete and to contain several doubtful entries.

## Introduction

Auroral activity is a well-known proxy for estimating the level of solar activity (e.g. Lee et al., 2004; Vázquez et al., 2016) and several articles have studied historical accounts of the aurora from specific geographical regions (e.g. Křivský and Pejml, 1988; Yau, Stephenson, and Willis, 1995; Basurah, 2010). As pointed out by Stangl and Foelsche (2020), the historical region of Transylvania, located in what is now Romania, has hitherto been somewhat neglected in historical studies regarding geophysical and astrophysical phenomena. Transylvania is situated in the southeastern Carpathian region in the heart of the European continent and was in the investigation period 1500 – 1800 CE part of the Kingdom of Hungary, or as a subordinate principality, part of the Ottoman Empire (1541 – 1699 CE). Source texts about historical events and natural phenomena from the period in question are mostly written in German or Latin, sometimes also in Hungarian and Romanian. The catalog of northern-light observations in Hungary 1523 – 1960 (Rethly and Berkes, 1963), which also includes the region of Transylvania, is also written in German. We discuss the entries available for our study period and provide selected text documents in English translation. The records that have already been known to science have been reexamined and are supplemented by 23 newly found records. Table 1 lists localities mentioned in the text, with their names in the three major spoken languages of the country and their approximate coordinates.

**Table 1:** List of localities mentioned in the text, given with their names in the three major spoken languages of the country and their approximate coordinates.

Romanian	Hungarian	German	Approx. coordinates
Bistrita	Beszterce	Bistritz	47°08′ N / 24°30′ E
Brasov	Braso	Kronstadt	45°40′ N / 25°37′ E
Cluj–Napoca	Kolozsvar	Klausenburg	46°46′ N / 23°35′ E
Codlea	Feketehalom	Zeiden	45°41′ N / 25°26′ E
Fantenele	Gyulakuta	Gielekonten	46°25′ N / 24°45′ E
Halchiu	Höltöveny	Heldsdorf	45°46′ N / 25°33′ E
Medias	Medgyes	Mediasch	46°10′ N / 24°21′ E
Prejmer	Prazsmar	Tartlau	45°43′ N / 25°46′ E
Rotbav	Veresmart	Rothbach	45°50′ N / 25°33′ E
Sibiu	Nagyszeben	Hermannstadt	45°48′ N / 24°09′ E
Sighisoara	Segesvar	Schäßburg	46°13′ N / 24°47′ E

## Historical Reports on Potential Northern Lights in Transylvania, 1501 – 1800 CE

The consulted sources from Transylvania are very sparse in mentioning natural phenomena predating the 16th century, and therefore we focused on the period from about 1500 CE onwards. Our first newly found record, i.e. unknown to Rethly and Berkes (1963), dates from the year 1593.

### 16th Century

Around the middle of the 16th century, the postulated Spörer minimum of solar activity came to an end. However, it is unclear to what extent the activity returned to a “normal” level in 11-year cycles of steadily increasing strength. In any case, an expected low number of sunspots and aurorae from that time is probably not the only reason for the lack of the historical records in Europe. With regard to Transylvania, there is simply a lack of sources in the number that characterizes the 17th century. Even though the number of reports on natural phenomena is increasing over the course of the 16th century, the earliest bona fide aurora in Transylvania dates from the year 1579. In the night from 8 February to 9 February “fire” was observed in the sky at Bistrita, in the North of the principality. A year later, in 1580, we learn, without specific location information, that “this year, the sky was seen three times completely on fire.” (Bielz, 1862). The compilation of historical northern lights by Pilgram (1788) lists the days 6 March, 6 and 9 April, 10 and 21 September, and 26 December for that year, without specifying the geographical region. After a two-year hiatus, on 20 August 1583, there was another phenomenon in the sky above Brasov, which Rethly and Berkes (1963) interpreted as northern lights. From the same year, on 5 September, after 1 in the morning, northern lights were seen in Bohemia and only faded at dawn (Křivský and Pejml, 1988).

In the Summer of 1591, “burning in the sky” was seen several times in Transylvania (Bielz, 1862). There are also reports from Northern Bohemia about potential aurora borealis on 8 September and 12 October: “On the evening of the Birth of Mary”, there was an intense auroral display, “like a blowing curtain, through which the stars were visible.” On 12 October, “two warriors fighting in the air” were reported (Křivský and Pejml, 1988). In the following year, 1592, reports were received from Rotbav in Transylvania’s Tara Barsei of fire signs in the sky that were visible all night on 29 December (Sutoris, 1903). According to Rethly and Berkes (1963), this phenomenon was also seen in Sighisoara. In the following January (1593), two aurorae seem to have been observed in Bohemia. However, the dramatic description of the former was embellished with all sorts of fantastic accessories, so it remains unclear what is actually true about the report. Another potential aurora was seen on 30 October (Křivský and Pejml, 1988). According to the historical report by Matthias Miles, Councilor of Sibiu, bright northern lights caused a sensation in Transylvania in the early morning hours of 12 January and in the nights of 28 and 30 November 28: “At the beginning of the year [1593], on January 12th, at 3 o’clock in the morning, a terrible burning in the sky was seen/also war servants, fighting with skewers and swords in the air; also high mountains were torn/so that in many places large churches and strong buildings sank beneath them: It proclaimed to all people the miserable condition/which was approaching Transylvania. [...] On November 28th, fiery skewers/which were fighting against each other/were seen in great numbers; also on the 30th day many terrible things and more/than before.” (Miles, 1670).

In 1599, on the evening of 28 May, at 8 in the evening, a red fire glow was seen in the sky in Brasov (Czack, 1909). Likewise, five days before the Battle of Schellenberg (today Selimbar, Judetul Sibiu) in the night from 23 to 24 October, the same thing was reported in Sibiu (Bethlen, 1785). In the night of 1 May 1600, at two places in Transylvania, in the eastern sky, “two armies fighting against each other” were seen (Krauss, 1839) and on 28 December, while an identical phenomenon was reported from Sighisoara.

We stress again that the lack of reports in the first decades of the century is by no means a strong indicator for missing auroral activity earlier in the 16th century, although a certain rising activity level towards the end of this century seems quite plausible.

### 17th Century

The new century was particularly rich in northern lights in its first two decades. Northern lights were seen several times in Bohemia in 1604 (Křivský and Pejml, 1988), and “at night a bloody rainbow has been seen” in Austria (Peinlich, 1878). “On the 29th day of March 1604, there was a great sign in the sky, so that it was fiery red from 4 to 6” (Banfi, 1909) and maybe even in other nights, because Georgius Krauss (Georg Kraus), city clerk from Sighisoara, reports that the sky in the North was stained with blood on several occasions (Krauss, 1839) In May, an aurora caused a sensation in Transylvania (Bethlen, 1793), and also in the night from 19 September to 20 September, when it was described as a terrible fire sign in the shape of a round war tent (Sepsi, 1858). This happened again in the early morning of 29 September (Veyss, 1909). Also, on 24 October, very striking northern lights were seen at various locations in Transylvania (Rethly and Berkes, 1963). “In 1604 fire rays were seen in the sky for 5 hours” is mentioned without a precise date by Nekesch-Schuller (1903).

The following year, 1605, represents the climax of reports about aurora borealis in the country. Accounts from no fewer than 13 nights with auroral phenomena have survived in various reports. However, only one of them is missing in the Rethly and Berkes (1963) collection, namely the report by Nösner from 3 November (see below). Interestingly, that year is also characterized by a presumably very powerful solar storm, as concluded by McCracken et al. (2001) based on ice-core analyses. On the evening of 6 June, the northern sky was seen blood-red in Transylvania (Krauss, 1839). However, the information is quite muddled, and it remains unclear whether it was indeed an auroral display. On the other hand, a second account from Transylvania (Veyss, 1909), dated 16 November, seems unquestionable. Likewise, another auroral display seems to have happened two weeks earlier: “On November 3, a figure of a great fiery man was seen in the sky.” (Nösner, 1903). Furthermore, aurorae from Brasov are reported from six nights in February, one in March, and two in December (Rethly and Berkes, 1963). The year 1605 is also rich in aurora reports from other parts of the world, especially Bohemia. “The 17th of November and the 20th of December [Northern Lights, according to] Serrarius, who reported them to [Johannes] Kepler,” writes Pilgram (1788). In the winter months of this year, northern lights were also seen in Korea, some of which were described in great detail (Yau, Stephenson, and Willis, 1995).

Two years later, in 1607, it is reported from Halchiu in Transylvania: “On January 18, the sky burned very brightly from 5 to 9 [p.m.]” (Nösner, 1903). Northern lights were also seen the same year on 17 November in Kaufbeuren in Bavaria (Pilgram, 1788). In the following years, aurorae were seen in Transylvania without interruption until 1617, for a total of 11 years. This strange fact suggests a particularly strong Sunspot Cycle −13, which corresponds to the cycle in which Galileo, Scheiner, and Harriot carried out their first telescopic solar observations. 1608, with apparent reference to the end of February, from Bistrita in Transylvania we simply learn: “This year the sky burned” (Bielz, 1862). A year later, on 8 September 1609 “[there was] seen bloodshed in the sky” in Cluj–Napoca, but also in other places in Transylvania (Rethly and Berkes, 1963).

The year 1610 is considered a minimum year in numerous reconstructions of early solar activity. Nevertheless, northern lights were seen in Transylvania in February and December (Rethly and Berkes, 1963). Likewise, in late Summer of the same year: “August 27th, 12 large fiery rays were seen in the sky for 4 hours.” (Forgats, 1903). A year later, in 1611, a bright aurora was seen at Sighisoara and Brasov on 27 August (Rethly and Berkes, 1963). In July 1612, an auroral display was noticed in Brasov (Segesvari, 1855), and in August “highly remarkable midnight signs, terrible to look at” are reported in Transylvania (Segesvari, 1855). Sighisoara’s former city clerk Georg(ius) Kraus(s) describes the display in detail, leaving little doubt that a strong, genuine aurora extended from the North towards the South: “A captain, who was sleeping at an open window because of the summer heat, saw terrible signs of flames in the sky at midnight: burning armies fighting each other in the western firmament and in the south there was a fire dragon with its maw wide open.” (Mioc and Mioc, 1977). There is also a more detailed description of this event: “When this [captain] came home a little drunk in the evening and laid down at midnight next to the window that went out to the field, he suddenly saw a terrible sign in the western sky, as if two burning armies collided and fought each other. Around midnight there was another sign in the form of a fiery dragon with its throat open. He was so frightened that he ran to [prince] Bathori that same night to tell him about the signs that had happened and to remind him that there was a righteous God. He asked him to let his evil intentions go and not to dip his hands in innocent blood.” (Kraus, 1864). In 1613, Bohemian chronicles reported on 12 October a “blood sign in the sky”. In Kuttenberg (today Kutna Hora, Czech Republic), one could see “bloody clouds in different places (of the sky) from all sides”. Another alleged aurora was seen in Bohemia half a year earlier, on 5 April (Křivský and Pejml, 1988). On 18 July, an auroral display was seen in Cluj and several northern lights in November were reported from Transylvania: 9/10, 10/11 (Segesvari, 1855), and 16/17; the latter two are said to have lasted all night until dawn (Rethly and Berkes, 1963). On 9th November it is reported: “In the evening, around 7 o’clock, the sky and the air in the whole Burzenland [Tara Barsei], especially over Brasso [Brasov], was blood-red, as if a rain of blood was ready to fall; it was mixed with white crowns and was black as coal in the lower part.” (Forgats, 1903). Also, “On the 16th, there was a great sign in the sky, very fiery, from 7 p.m. to 4 a.m. all night.” (Banfi, 1909).

On 20 and 23 February 1614, northern lights were seen at a non-specified location in Transylvania; the former was perceived as an amorphous glow of fire around midnight (Massa, 1903), while the latter formed clearly visible stripes: “On February 23rd there was a great sign in the sky with fiery red marks.” (Banfi, 1909). From the following year, 1615, which according to sunspot reconstructions likely represented the maximum year of Cycle −13, there is the following note from the principality: “On August 30th, large signs were seen on the firmament, large armies, large projectiles were heard, and many riders apparently heard and seen.” (Forgats, 1903). Also, according to Pilgram (1788): “On the 26th of October fiery men fought in the sky.” In 1616, on 10 April, a “white and red sign in the night sky” was seen in Transylvania (Sepsi, 1858), although (as in so many other cases) it remains unclear whether it was a genuine auroral display or not, due to the lack of detail in the description. An aurora seems to have been seen in Bohemia in February 1617 (Křivský and Pejml, 1988). A celestial sign in Transylvania that was seen on 30 August also suggests an auroral display, although this is said to have been accompanied by sounds: On 30 August, “great signs were also seen on the firmament, great armies, great shooting heard [and] many riders were apparently heard and seen.” (Forgats, 1903).

This series of 11 years, in which aurorae have been seen every year, is followed by a period of 12 years, in which we have no relevant news from the Principality. It was not until the evening of 13 February 1629 that northern lights seem to have been seen in the area of Bistrita; the rays of the auroral crown were compared to horsetails (Eber, 1559). After another long hiatus of 13 years, on 4 January 1642, apparently another aurora appeared over Fantanele in today’s Mures district. However, the report mentions a violent noise when the red and the white “army crashed into each other” (Rethly and Berkes, 1963). The erroneous mentioning of sounds accompanying aurorae is curiously widespread in the old literature and, in our opinion, should be interpreted and accepted as a fantasy embellishment of the records in order to attract the reader. From the following year, 1643, the Transylvanian chronicler Georg(ius) Kraus(s) reports: “On January 5, a large heavenly sign, a brightly burning fire as wide as a table, was also seen here in Transylvania, in the old country and elsewhere, which finally ejected fiery rays, and disappeared with a big clap of thunder; it was seen both by day and by night; what the consequence may be, will be seen only with time passing.” (Kraus, 1864). However, this report is very confused and should, in our opinion, not be interpreted as an aurora. Most likely, a meteor may have been seen, or various celestial phenomena have been mixed together.

Two years later, the Maunder minimum, the best-known and best-documented occurence of a grand minimum of solar activity, started. Nevertheless, even during this time of drastically reduced solar activity, reports of suspected aurorae at low geographic latitudes are by no means lacking. Transylvania is situated around 46° North and is therefore a good indicator for an aurora oval extending unusually far towards the Equator. Nine reports from Transylvania between 1661 and 1713, five of which are listed by Rethly and Berkes (1963), suggest that auroral activity in the period in question did not weaken as completely as generally assumed. Of these nine possible northern lights, we believe seven to be genuine aurorae and two to be bright meteors.

In 1661, on 23 April, late in the evening, “columns of fire” and “fiery clouds” were seen in the sky over London (Vallee and Aubeck, 2010). In the evening of 10 September, the sky was said to be “burning” over Sighisoara in Transylvania, as if all the towers of the whole city were on fire: “... which day at 5 o’clock in the evening, a terrible sign in the sky was visible when it appeared very fiery, and for an hour it shone on the towers and [the] city as if everything was on fire, which I am truthfully writing because many opinions have been proclaimed, Deus misereatur nobis.” (Kraus, 1864).

In 1662, in the night from 23 to 24 February, in Sibiu, Transylvania, two “flaming armies” appeared in the sky, fighting against each other (Kraus, 1864). In 1684, in the late evening of 8 October, in Mediasch (Medias) in Transylvania, a celestial phenomenon was seen that was very likely a genuine auroral display (Rethly and Berkes, 1963). 1687, “in July, northern lights were observed by the great Cassini.” (Pilgram, 1788). In the same month, on 13 July, either a meteor or an aurora was seen above Sibiu in Transylvania (Bielz, 1862). In 1693, in an unknown location in the principality, on “February 24th, various signs such as burning lights, rumbling and fiery dragons were seen at night.” (Seyberger and Blasius, 1918).

### 18th Century

In the last decade of the Maunder minimum, the Sun seems to have recovered gradually, as indicated by studies of various proxies (Vaquero and Trigo, 2015). Therefore, it comes as no surprise that reports of aurorae were also starting to accumulate in Transylvania. In 1704 “northern lights were seen against the south of the sky.” (Pilgram, 1788). A fiery sign was seen on the sky in Transylvania’s Tara Barsei, which might have been a genuine aurora: “On June 21 there was a fiery sign between 11 and 12 o’clock flying over the city at night.” (Seyberger and Blasius, 1918). A similar phenomenon was seen in Cluj on 1 August (Czegei-Vass and Czegei-Vass, 1893). Northern lights were also seen in Hamburg in Germany this year (Vallee and Aubeck, 2010).

On two nights in March 1709, the sky in Bohemia was brightened up at 10 in the evening, which lasted three-quarters of an hour each (Křivský and Pejml, 1988). Already in the month before, northern lights from Transylvania have been described as bright and lasting for three hours; aurorae were also reported from Berlin and Copenhagen at the time (Mioc and Mioc, 1977). In 1713 another aurora was seen at a not-specified location in Transylvania, but the exact date has not been preserved (Rethly and Berkes, 1963).

In the years after the end of the Maunder minimum, an increase in auroral observations can be found in Europe and worldwide. However, it remains unclear to what extent it is actually a clustering of aurorae or just an artifact, as European scientists started to focus on the phenomenon. According to Pilgram (1788), several northern lights were seen in 1719. In Transylvania, an aurora is mentioned shortly before Christmas (25 December): “On December 22nd at midnight, a strong fire-like reddening is seen in the air.” (Teutsch, 1903a). In Transylvania there follows a period of 14 years without any known auroral sightings. In 1734, the next year in which Transylvania contributed an observation, many northern lights have been reported across Europe, however: “One of these northern lights stood in the midday region of the sky.” i.e. in the South, as Pilgram (1788) reports, which makes its reality somewhat doubtful. In Transylvania, an auroral display was seen on 25 January 1734 (Apor, 1863). Two years later, on 20 October 1736, in Prejmer in Transylvania, “great” northern lights have been seen between 8 and 11 p.m. (Tartler, 1903). In the following year, 1737, on 16 December, Europe saw “great northern lights” down to Lisbon (Fritz, 1873). Between 9 and 10 at night. the sky over Bohemia was so bright that the landscape was “as if bathed in blood.” (Křivský and Pejml, 1988). “In the memory of men no one seemed so great”, writes Pilgram (1788). The phenomenon mentioned above was also seen in Transylvania’s Tara Barsei region. The Annals of Codlea noted: “... on December 16, at 8 o’clock in the evening, a scary redness was perceived in the firmament, as if villages were burning, white spaces in between; it lasted until around midnight; afterward, one part went towards the sunrise, the other towards sunset; this lasted until the second hour of the morning.” (Annales Czeidinenses, 1909). Already ten days before that: “1737, the 6th of December, at 9 p.m. with otherwise clear skies, a fiery glow appeared in the direction of midnight [i.e. in the North], from which short and long rays rose, some of which extended to the North Star, so that everything remained bright in the air until morning.” (Teutsch, 1903a; Mioc and Mioc, 1977). Also, in China northern lights were seen and described in detail on the same day (Yau, Stephenson, and Willis, 1995). Two weeks later, southern lights, which “made the mountains glow”, have been seen on the Chilean island of Chiloe (Fritz, 1873). Northern lights reported from Transylvania for 1738, may actually refer to the event of the previous year just discussed, because the date is identical to the spectacular appearance of the previous year: “It is also on December 16, when at 8 o’clock in the evening, a terrible redness in the firmament was seen around midnight, as if villages had been set on fire, and was divided up with white streams; [it] lasted until midnight; afterward, one part split up towards morning [i.e. to the East], the other part towards evening [i.e. to the West]; [it] lasted until after midnight at 2 o’clock.” (Czack, 1909).

On 28 October 1768, “red clouds and pillars” were seen in the evening sky in Bohemia. (Křivský and Pejml, 1988). The same phenomenon was also perceived in Transylvania (Teutsch, 1903b). A few days earlier, an aurora in the form of a crown was seen in neighboring Moldova (Mioc and Mioc, 1977). In 1779, “In this year up to March 25th [...] for six times, strong celestial signs with great redness like clouds of fire were seen” in Bohemia. (Křivský and Pejml, 1988). Pilgram (1788) reports observations made by himself from Vienna: “This year I observed four northern lights here. February 13th, March 14th, November 9th, December 5th.” On 18 September, an aurora was reported from Transylvania. (Dück, 1903).

In 1788, northern lights were seen as far South as Spain and southern Italy (Fritz, 1873). On 5 November, there was an auroral display over Bohemia (Křivský and Pejml, 1988). At the beginning of September, several aurorae, shining intensely red, have been reported in Transylvania and neighboring Moldova (Mioc and Mioc, 1977) and “On the day of the 19th in the evening at 9 o’clock great northern lights.” (Czack, 1909). Finally, in 1790, a robust auroral display was seen in Codlea near Brasov in Transylvania: “In 1790 on January 29, very great northern lights seen in the evening at 6 o’clock.” (Czack, 1909).

## Compilation of an Aurora-Table

Table 2 shows the essential information about the 78 possible auroral displays between 1500 and 1800 CE observed from Transylvania that have been presented here in detail. The first column gives the date according to the Gregorian calendar. The second column lists the place of observation with its current name (city or region) in the eastern European state of Romania. The third column indicates whether the sighting in question is listed by Rethly and Berkes (1963). The fourth column shows the degree of probability of whether the phenomenon was a genuine aurora. Columns 5 to 8 list the number of entries in the auroral catalogs by Schröder (1992) “Schr”, Křivský and Pejml (1988) “K/P”, Yau, Stephenson, and Willis (1995) “Yau”, and Lee et al. (2004) “Lee.” The last column gives a brief comment on the presumed solar activity of the calendar year in question.

**Table 2:** Main information about the 78 possible auroral displays between 1500 and 1800 CE observed from Transylvania that have been presented here in detail. The first column gives the date according to the Gregorian calendar. The second column lists the place of observation with its current name (city or region) in the East European state of Romania. The third column indicates whether the sighting in question is listed by Rethly and Berkes (1963). The fourth column shows the degree of probability of whether the phenomenon was a genuine aurora. Columns 5 to 8 list the number of entries in the auroral catalogs by Schröder (1992) “Schr,” Křivský and Pejml (1988) “K/P,” Yau, Stephenson, and Willis (1995) “Yau”, and Lee et al. (2004) “Lee.” The last column gives a brief comment on the presumed solar activity of the calendar year in question.

Aurorae sightings from Transylvania, 16th – 18th century								
Date	Locality	R/B entry	Reliability	Number of entries for year				Remarks on solar activity
				Schr.	K/P	Yau	Lee	
1579 Feb. 8	Bistrita	yes	high	0	2	0	0	Recovering from Sporer minimum
1579 Feb. 9	Bistrita	yes	high	0	2	0	0	Recovering from Sporer minimum
1580	Sibiu	yes	high	10	11	0	0	Recovering from Sporer minimum
1580	Sibiu	yes	high	10	11	0	0	Recovering from Sporer minimum
1580	Sibiu	yes	high	10	11	0	0	Recovering from Sporer minimum
1583 Aug. 20	Brasov	yes	low	7	10	3	0	Recovering from Sporer minimum
1591 Summer	not specified	yes	very high	3	3	1	1	Possible maximum year
1592 Dec. 29	several	yes	very high	3	3	0	0	-
1593 Jan. 12	several	yes	very high	4	6	1	1	-
1593 Oct. 28	Sighisoara	yes	very high	4	6	1	1	-
1593 Nov. 30	not specified	no	very high	4	6	1	1	-
1599 May 28	Brasov	yes	high	5	5	2	2	Supposed solar storm
1599 Oct. 23 or 24	Sibiu	yes	very high	5	5	2	2	Supposed solar storm
1600 May 01	not specified	no	high	2	2	0	0	-
1600 Dec. 28	Sighisoara	yes	high	2	2	0	0	-
1602 Nov. 04	Brasov	yes	low	2	3	1	1	-
1604 May	not specified	no	high	9	13	2	3	-
1604 Sep. 19/20	Sighisoara	no	high	9	13	2	3	-
1604 Sep. 29	several	yes	very high	9	13	2	3	-
1604 Oct. 24	several	yes	high	9	13	2	3	-
1605 Feb. 09	Brasov	yes	high	4	14	5	5	Supposed very strong solar storm
1605 Feb. 15	Brasov	yes	high	4	14	5	5	Supposed very strong solar storm
1605 Feb. 16	Brasov	yes	high	4	14	5	5	Supposed very strong solar storm
1605 Feb. 17	Brasov	yes	high	4	14	5	5	Supposed very strong solar storm
1605 Feb. 18	Brasov	yes	high	4	14	5	5	Supposed very strong solar storm
1605 Feb. 19	Brasov	yes	high	4	14	5	5	Supposed very strong solar storm
1605 Mar. 22	Brasov	yes	high	4	14	5	5	Supposed very strong solar storm
1605 Jun. 06	Sighisoara	yes	high	4	14	5	5	Supposed very strong solar storm
1605 Nov. 03	not specified	no	high	4	14	5	5	Supposed very strong solar storm
1605 Nov. 16	Brasov	yes	high	4	14	5	5	Supposed very strong solar storm
1605 Dec. 17	Brasov	yes	high	4	14	5	5	Supposed very strong solar storm
1605 Dec. 18	Brasov	yes	high	4	14	5	5	Supposed very strong solar storm
1607 Jan. 18	Halchiu	yes	high	2	3	0	0	Sunspot seen by Kepler
1608 Feb., end of	Bistrita	yes	medium	1	3	0	2	Possible maximum year
1609 Sept. 08	several	yes	very high	1	3	0	0	-
1610 Feb.	several	yes	high	1	2	0	2	Probable minimum year
1610 Aug. 27	not specified	no	very high	1	2	0	2	Probable minimum year
1610 Dec. 17	not specified	yes	very high	1	2	0	2	Probable minimum year
1611 Aug. 27	several	yes	very high 1	2	1	2	-	
1612 Jul. 11	Brasov	yes	low	1	5	0	0	Supposed solar storm
1612 Aug.	Sighisoara	no	very high	1	5	0	0	Supposed solar storm
1612 Aug. 04	Cluj	yes	high	1	5	0	0	Supposed solar storm
1612 Aug. 28	Cluj	yes	very high	1	5	0	0	Supposed solar storm
1613 July 18	Cluj	yes	high	3	7	1	1	-
1613 Nov. 09	Tara Barsei	yes	high	3	7	1	1	-
1613 Nov. 10	Cluj	yes	medium	3	7	1	1	-
1613 Nov. 12	Brasov	yes	medium	3	7	1	1	-
1613 Nov. 13	Brasov	yes	high	3	7	1	1	-
1614 Feb. 20	several	yes	very high	1	4	0	0	-
1614 Feb. 23	Brasov	yes	medium	1	4	0	0	-
1615 Aug. 30	not specified	no	high	1	6	0	0	-
1615 Dec. 27	Brasov	yes	very low	1	6	0	0	-
1616 Apr. 10	not specified	no	high	1	1	0	0	Supposed solar storm
1617 Aug. 30	not specified	yes	medium	0	2	0	3	
1629 Feb. 13	Bistrita	no	high	9	11	1	1	-
1642 Jan. 04	Fantanele	yes	medium	2	2	0	0	-
1643 Jan. 05	Sighisoara	no	low	2	3	1	0	
1661 Sep. 10	Sighisoara	no	high	4	6	0	0	Maunder minimum
1662 Feb. 23/24	Sibiu	no	very high	3	3	0	0	Maunder minimum
1684 Oct. 08	Médias	yes	medium	1	1	0	0	Maunder minimum
1687 Jul. 13	Sibiu	yes	very low	2	2	1	0	Maunder minimum
1693 Feb. 24	not specified	no	medium	1	0	1	1	Maunder minimum
1704 Jun. 10	not specified	no	very low	4	7	0	0	Maunder minimum
1704 Aug. 01	Cluj	yes	high	4	7	0	0	Maunder minimum
1709 Feb.	not specified	no	very high	2	2	0	0	Maunder minimum
1713 Jan.	not specified	yes	medium	1	3	1	1	Maunder minimum
1719 Dec. 23	several	yes	high	8	29	0	0	-
1730 Feb. 14	Brasov	yes	low	-	50	3	2	Supposed solar storm
1730 Feb. 15	several	yes	very high	-	50	3	2	Supposed solar storm
1734 Jan. 25	not specified	yes	medium	-	24	0	0	-
1736 Oct. 20	Prejmer	no	high	-	37	0	0	-
1737 Dec. 16	several	yes	very high	-	38	1	0	-
1768 Oct. 28	not specified	no	very high	-	7	0	-	-
1779 Sep. 18	not specified	no	high	-	72	-	-	-
1788 Aug. 19	Codlea	no	high	-	75	-	-	-
1788 Sep., beg. of	not specified	no	high	-	75	-	-	-
1788 Sep. 19	not specified	no	high	-	75	-	-	-
1790 Jan. 29	Codlea	no	high	-	42	-	-	Dalton minimum

The evaluation of the degree of probability of a genuine auroral sighting must necessarily remain subjective due to the material available. Based on the specified text passages or their summary here and in Rethly and Berkes (1963), researchers are able to verify what the chronicler actually reported. Depending on how strictly the screening is applied, more or fewer real aurorae will be rejected along with erroneous ones. Here we tried to take a middle course: If it is clearly stated or recognizable that the phenomenon appeared at night, if the usually conspicuous structures and red coloring have been noted, if the duration of the event indicates several hours, if the phenomenon has been called *expressis verbis* “northern light” by the chronicler or if the contemporary, sometimes fancifully embellished interpretation as “armies in the sky” is stated, it was assumed that the probability was “medium” to “very high”. If, on the other hand, too many circumstances remain unclear or if the description suggests, for various reasons, that the date is in error or that the appearance was likely mistaken for meteors or atmospheric phenomena, the labels “low” or “very low probability” were assigned. Attached suspicious information, such as sounds allegedly accompanying the phenomenon, reduces the likelihood of an otherwise almost unambiguous report.

**Figure 1:**
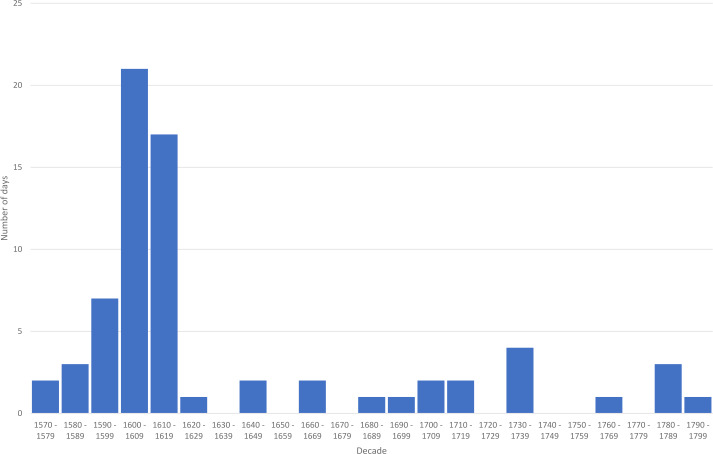
Number of known auroral sightings from the former principality of Transylvania within the Kingdom of Hungary in the decades between 1570 and 1799 CE. Only observations of “medium” to “very high” probability are counted.

**Figure 2:**
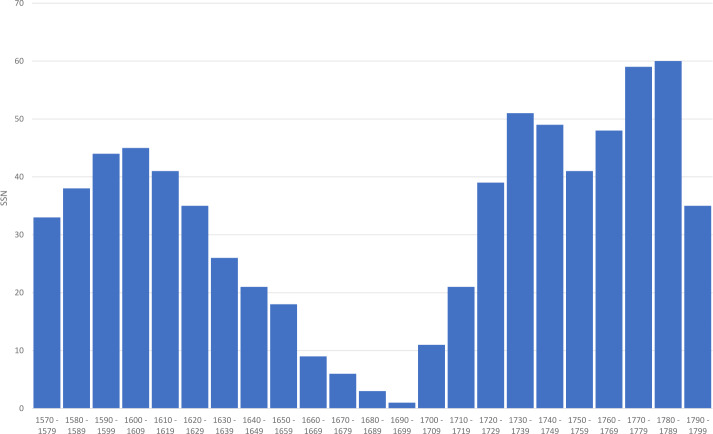
Decadal Sunspot Numbers (SSN) in rounded mean values, reconstructed from ^14^C and ^10^Be proxy data, according to Wu et al. (2018), for the decades between 1570 and 1799 CE.

Figure 1 shows the compiled number of known aurorae from Transylvania, with assigned probabilities from “medium” to “very high”. It is to be compared with Figure 2, which shows the estimated sunspot number (rounded values) based on proxy data (carbon-14, beryllium-10), according to Wu et al. (2018). Both values are calculated per decade (total number of aurorae and mean sunspot number). While we generally understand centuries and decades as starting with the year “1”, there is an alternative counting that defines decades from the 0 years to the years ending with 9. Since the best available reconstruction of long-past solar activity by Wu et al. (2018) uses the latter and we compare our auroral reports with them, we preferred this scheme over the other in the interest of better compatibility between Figure 1 and Figure 2.

Although the agreement is quite reasonable for the two decades 1601 – 1610 and 1611 – 1620, it clearly shows that the high solar activity beginning with the 1720s does not go hand in hand with an increase in the auroral frequency in Transylvania after the end of the Maunder minimum. However, we conclude that selective perception and a shift in the focus of interest over decades and centuries did have a more considerable influence on the records than the actual frequency or manifestation of auroral displays.

## Source Criticism and Discussion

Neuhäuser, Neuhäuser, and Chapman (2018) have designed a five-point scheme aiming to distinguish genuine auroral observations from false ones, i.e. confusion with atmospheric phenomena, comets, meteors, etc. However, this scheme assumes that the phenomenon has been not only meticulously observed, but also recorded and reported in some kind of scientific manner. Undoubtedly, this was not the case for the investigation period. These criteria are, therefore, not applicable in our case. As mentioned above, we refrained from providing numerical indices about the reliability of the listed observations in Table 1. Instead, a five-step verbal assessment was used, from “very high” to “very low” probability. Figure 2 shows the estimated solar-activity curve according to the reconstruction by Wu et al. (2018) and links it with the number of reported northern lights from Transylvania (Figure 1); only entries with a probability level of “medium” or higher were taken into account. The comparison shows that the clustering of known potential auroral descriptions from Transylvania correlates poorly with solar activity as reconstructed from proxy data. This is to be interpreted as a sign of selective tradition, as is rather the rule for historical sources of the time, especially within a war-stricken province during the great battles between the Ottoman Empire and the western world, i.e. in the case of these reports, the focus was not on exact scientific description, but on emphasizing the extraordinary and also often on the opportunity of use for purposes of political or religious propaganda. It is further noticeable that in the catalog of Rethly and Berkes (1963), almost all of the auroral sightings between 1579 and 1614 come from Transylvania, namely 39 out of 41, i.e. 95%, and 36 out of 41 (88%) if one counts parallel sightings from other parts of the Kingdom of Hungary. It is clear that this situation reflects different degrees of accessibility and availability of certain sources at certain times. The assumption by Vaquero and Trigo (2015): “the series of auroras observed in Hungary [...] appear to ensure better uniformity and a more straightforward link to solar activity”, and thus the assumed higher reliability of the catalog by Rethly and Berkes (1963) as compared to the one compiled by Křivský and Pejml (1988) cannot be supported. The sighting of seven genuine northern lights with “medium” to “very high” probability in Transylvania during the Maunder minimum (in the years 1661, 1662, 1684, 1693, 1704, 1709, and 1713) suggests that from time to time aurorae have extended into geographical latitudes of around +45° even during this period of significantly reduced solar activity.

## Summary and Conclusions

We were able to discuss in detail and present translations of eyewitness accounts regarding 78 possible auroral sightings for the period 1501 – 1800 CE from today’s Romanian province of Transylvania, then part of the Kingdom of Hungary. 23 potential aurorae have been identified, which are missing in the catalog by Rethly and Berkes (1963) and are introduced here for the first time into the scientific discourse on past solar activity. In the second half of the 18th century all six entries that we found are missing from the compilation of Rethly and Berkes (1963), who do not list any sightings from Transylvania at all for that period. Located at roughly 46° northern latitude, the selected area is a good geographical indicator for an auroral oval extending unusually far towards the Equator. The reports of seven celestial phenomena from Transylvania during the period of the Maunder minimum (1645 – 1715), which we consider genuine northern lights at a medium to very high probability level, suggest that even during a truly grand minimum of solar activity, auroral activity was strong enough to penetrate down at least to near 45° latitude, from time to time. Three of these potential aurorae, however, fall within the 18th century, when the Sun was already recovering from the deep minimum phase. The distribution of potential auroral sightings from Transylvania during the studied period, i.e. 1501 – 1800, clearly shows, compared with other aurora catalogs and with reconstructed solar activity, that high selectivity due to the historical-source situation makes statements about actual long-term distributions almost impossible.
